# Health and social exclusion in older age: evidence from Understanding Society, the UK household longitudinal study

**DOI:** 10.1136/jech-2016-208037

**Published:** 2017-02-22

**Authors:** Amanda Sacker, Andy Ross, Catherine A MacLeod, Gopal Netuveli, Gill Windle

**Affiliations:** 1 Department of Epidemiology and Public Health, University College London, London, UK; 2 Dementia Services Development Centre Wales, Bangor University, Bangor, UK; 3 Institute for Health and Human Development, University of East London, London, UK

**Keywords:** SELF-RATED HEALTH, SOCIAL EPIDEMIOLOGY, MENTAL HEALTH, LONGITUDINAL STUDIES, ELDERLY

## Abstract

**Background:**

Social exclusion of the elderly is a key policy focus but evidence on the processes linking health and social exclusion is hampered by the variety of ways that health is used in social exclusion research. We investigated longitudinal associations between health and social exclusion using an analytical framework that did not conflate them.

**Methods:**

Data employed in this study came from 4 waves of Understanding Society, the UK Household Longitudinal Study 2009–2013. The sample comprised all adults who took part in all 4 waves, were 65 years or more in Wave 3, and had complete data on our variables of interest for each analysis. We used linear regression to model the relationship between Wave 2/3 social exclusion and Wave1–2 health transitions (N=4312) and logistic regression to model the relationship between Wave2/3 social exclusion and Wave 4 health states, conditional on Wave 3 health (N=4244).

**Results:**

There was a dose–response relationship between poor health in Waves 1 and 2 and later social exclusion. Use of a car, mobile phone and the internet moderated the association between poor health and social exclusion. Given the health status in Wave 3, those who were more socially excluded had poorer outcomes on each of the three domains of health in Wave 4.

**Conclusions:**

Use of the internet and technology protected older adults in poor health from social exclusion. Age-friendly hardware and software design might have public health benefits.

## Introduction

Social exclusion is a multidimensional process through which individuals become disengaged from mainstream society, depriving people of the rights, resources and services available to the majority.^1^ A key priority for policymakers in Europe,^2^ social exclusion manifests through a number of interlinked and mutually reinforcing problems that deny people the opportunities available to most in society. There are a number of drivers of social exclusion including poverty, lower levels of educational attainment, unemployment, ill health, poor housing or homelessness, poor transport access, increased levels of crime and limited social support, all of which can have long-lasting effects.^3^ The interlinked nature of social exclusion makes it difficult to understand the relationships between differing domains, and to tease apart those that are direct risk factors, mediating or moderating factors, indicators or outcomes of exclusion.^4^ This complex relationship can clearly be seen with the health domain, where poor health is often considered a predictor or risk factor,^5–7^ an indicator^8–10^ or an outcome of exclusion.^6^ ^11–13^ The variety of ways that health is used in social exclusion research, and the many pathways through which social exclusion and health interact,^14^ constrain our understanding of the process and consequently possible solutions to resultant health inequality.

As people grow older, the chance that they will become socially excluded is greater than the chance that they will move out of or become less excluded.^7^ This highlights the severity and continuity of social exclusion for older adults. Current healthy ageing strategies in Europe are designed to try to address issues including social exclusion,^15^ by providing an environment in which people can engage in a process of ‘active ageing’, allowing them to “realize their potential for physical, social and mental well-being throughout the life course and to participate in society according to their needs, desires and capacities, while providing them with adequate protection, security and care when they require assistance”.(ref. 16 p. 12) While such policies are clearly designed to reduce the chances of people becoming socially excluded, the current lack of understanding about the pathways and mechanisms through which social exclusion exists is likely to inhibit their overall success.

Using 4 waves of data from a large UK household panel survey,^17^ we explore the process of social exclusion in later life. With health being a particularly important correlate of social exclusion for older adults, this paper focuses on the association between health and social exclusion, examining (1) whether poor health is a predictor of social exclusion in people aged 65 years and over; (2) whether health is an outcome of social exclusion; and (3) factors that might modify these relationships.

## Methods

### Participants

Data come from the first four waves of Understanding Society, the UK Household Longitudinal Study (UKHLS).^17^ The UKHLS is a nationally representative study, which began in 2009 with an aim of recruiting over 100 000 individuals in 40 000 households. The data collection period takes 2 years to complete one wave of the study. All persons in the household aged 10 years and older are eligible to be surveyed annually. Adults, 16 and older, are offered a combination of computer-assisted personal interview and self-completion questionnaire. After July 2012, computer-assisted telephone interviews were offered to non-responders. The topics covered include subjective well-being, employment, health and various other economic and social topics. More detailed information on the sampling frame and data collection procedures are available.^18^

The sample for our study includes members of the general population sample of Understanding Society who took part in Waves 1–4 and was aged 65 years or more in Wave 3. Of the 6473 aged 65+ in Wave 3, 5475 were interviewed in each of the first four waves. Item non-response reduced the sample to 4312 and 4244 for research questions 1 and 2, respectively. Online supplementary tables A1 and A2 in the online appendix show that the analysis samples were more advantaged and in better health than the samples of excluded respondents.

10.1136/jech-2016-208037.supp1 supplementary appendix

### Measures

#### Social exclusion

Following Walsh *et al*,^19^ a social exclusion index was constructed with three underlying domains: (1) Service provision and access; (2) Civic participation; and (3) Social relations and resources. Each subdomain comprised 4–5 indicators capturing relevant aspects of social exclusion pertaining to that domain. The guiding principle for the selection and construction of these indicators was that each should identify the most excluded quartile of individuals.

To overcome the problem that not all indicators of social exclusion were available in the same wave, social exclusion was measured using data from two consecutive waves of Understanding Society (Wave 2 (2010/2011) and Wave 3 (2011/2012)). A summary of the indicators and methods used for constructing social exclusion is given below. For a more in-depth overview, see MacLeod *et al*.^1^

*Service provision and access:* respondents were allocated a point for each of the following: reporting that they were not able to access all services such as healthcare, food shops or learning facilities when they needed to; rating the quality of local medical facilities as ‘fair’ or ‘poor’; rating local shopping facilities as ‘fair’ or ‘poor’; rating local leisure facilities as ‘poor’; and/or reporting that they found it ‘difficult’ or ‘very difficult’ to get to a sports or leisure facility if they wanted to, including a leisure centre, recreation ground or park. Scores were summed to give an overall scale from 0 to 5 with high scores indicating poorer service provision and access.

*Civic participation:* Respondents identified activities they had participated in during the past 12 months from predefined lists of cultural, sport and leisure activities, and reported the frequency with which they participated in each set of activities. Two items were derived to give the breadth (number of activities) and frequency of participation. Respondents scored a point for each indicator where they were in the bottom quartile. Respondents were also allocated 1 point for not regularly participating in the work of an organisation or group (from 16 listed organisations), and 1 point if they did not volunteer. Scores on the 4 items were summed and recalibrated to give an overall scale from 0 to 5 with high scores indicating poorer civic participation.

*Social relations and resources*: Respondents who lived alone were allocated 2 points, and respondents living with a spouse or partner were allocated 1 point if they scored within the bottom quartile of a relationship closeness scale. Respondents were allocated 1 point if they did not have a child living outside of the home or their level of contact with that child was especially low. One point was allocated if the respondent reported having one or no close friendships, and 1 point if they reported not going out socially or visiting friends when they felt like it. Scores were summed to give an overall scale from 0-5 with high scores indicating poorer social relations and resources.

The social exclusion index was derived by summing scores for the three subdomains, measured on a scale of 0–15 with higher scores indicating greater social exclusion.

Prior to construction of the social exclusion subdomains, imputation using chained equations (ICE) was employed to impute missing values if respondents were missing a single item within a subdomain.^20–22^ The table in MacLeod *et al*^1^ shows the prevalence for indicators preimputation and postimputation.

#### Health

Health measures include poor self-rated health (SRH: excellent, very good, good vs fair or poor); limiting long-term illness or disability (LLTI: no vs yes); and psychological distress, measured using General Health Questionnaire (GHQ) with the bimodal scoring method and a cut-off of 3 or more signifying distress (no vs yes).^23^ ^24^ Derived health transition variables (Wave 1 to Wave 2) took values 0 stable good; 1 declining; 2 improving; and 3 stable poor health.

#### Covariates

Covariates were split into two groups: (1) confounders and (2) mediators and/or modifiers.

*Confounders:* gender;^25^ age; age-squared;^26^ ethnicity (White or non-White);^27^ ^28^ place of birth (born in the UK or elsewhere);^27^ ^28^ marital status (married/in civil partnership, living as a couple, single never married/in civil partnership, separated or divorced, or widowed);^25^ job status (whether the respondent was in work or not);^29^ highest qualification (degree, other higher, A level or equivalent, GCSE or equivalent, other, or no qualifications);^28^ ^30^ social class (NS-SEC managerial and professional, intermediate, small employer and own account, lower supervisor and technical, semiroutine and routine occupations, or whether the respondent never had a job);^26^ ^30^ region (whether the respondent lived in one of nine Government Office Regions of England, or in Scotland, Wales or Northern Ireland).^31^

*Mediators/moderators:* Potential modification of the health and social exclusion association was assessed in relation to: area type (rural/urban);^14^ ^32^ ^33^ car access (whether the respondent lived in a household that owns or has continuous use of a car or not);^34^ ^35^ mobile phone ownership, and internet use (whether the respondent used the internet often (daily or several times a week), sometimes (several times a month or less), or never (never used it or no access at home, work or elsewhere).^36^

### Data analysis

The effect of health transitions on social exclusion was assessed using linear regression. Four models were estimated for each of the three health transition measures examined (SRH, LLTI, GHQ): (1) Base model adjusting for gender, age and age-squared; (2) further adjustment for ethnicity, country of birth, marital status, job status, highest qualification, social class and region; (3) further adjustment for area type, car access, mobile phone ownership and internet use; and (4) further adjustment for the remaining two health measures. Social exclusion was measured in Waves 2 and 3 of Understanding Society, health was measured over Waves 1 to 2 and all other measures were from Wave 1. The gender invariance of the association between health transitions and social exclusion was assessed using an interaction term in model 1. Since we found no evidence of an interaction between the health transitions and gender, results are not stratified. Modification of the relation by area type, car access, mobile phone ownership and internet use was assessed by adding interaction terms to model 4.

Second, the effect of social exclusion on subsequent health was assessed using logistic regression. Three models were estimated for each of the three health measures examined: (1) Base model adjusting for gender, age and age-squared and all three health measures; (2) further adjustment for ethnicity, country of birth, marital status, job status, highest qualification, social class and region; (3) further adjustment for area type, car access, mobile phone ownership and internet use. The health outcomes were measured in Wave 4 and social exclusion in Waves 2 and 3. Time-invariant measures (ethnicity, country of birth, highest qualification) were measured in Wave 1 and time-varying measures (health, marital status, job status, social class and region in Wave 3. The gender invariance of the association between social exclusion and health was assessed using an interaction term in model 1: no evidence of interactions between social exclusion and gender were found. Modification of the relation by area type, car access, mobile phone ownership and internet use was assessed by adding interaction terms to model 3.

All analyses used survey methods in Stata V14.1(Stata Statistical Software: Release 14.1 [program]. College Station, Texas: StataCorp LP., 2015) to provide cluster-robust SEs and Wave 4 longitudinal weights applied to take account of unequal selection probabilities, attrition and the non-response of eligible participants.

Further models assessed whether the association between health transitions and social exclusion, and social exclusion and subsequent health, varied across the three subdomains of social exclusion (service provision and access, civic participation and social relations and resources).

## Results

### Social exclusion and prior health

Table 1 shows mean differences in the social exclusion index (SEI) by the health and covariate measures. Mean SEI was of a similar magnitude irrespective of the health measure, ranging from around 4 for those in stable good health to almost 6 for those in stable poor health. SEI means also differed across values of the covariates in expected directions with the exception of area type; there were no urban/rural differences in SEI.

**Table 1:** Mean SEI and 95% CIs at Wave 2/3 by explanatory factors (N=4312)

	SEI	
Explanatory factor	Mean	95% CI
**Wave 1/2**		
*SRH*		
Stable good	3.80	3.69 to 3.91
Good→poor	4.99***	4.68 to 5.30
Poor→good	4.93***	4.61 to 5.25
Stable poor	5.75***	5.54 to 6.96
LLTI		
Stable no LLTI	3.83	3.72 to 3.95
LLTI onset	4.50***	4.19 to 4.82
LLTI recovery	4.47***	4.20 to 4.73
Stable LLTI	5.34***	5.16 to 5.51
GHQ		
Stable low	4.13	4.02 to 4.24
Low→high	4.99***	4.68 to 5.30
High→low	4.86***	4.54 to 5.17
Stable high	5.87***	5.54 to 6.19
Wave 1		
*Gender*		
Female	4.60	4.47 to 4.72
Male	4.20***	4.08 to 4.32
Age (years)		
<75	4.04	3.94 to 4.14
≥75	5.19***	4.99 to 5.39
Ethnicity		
White	4.40	4.30 to 4.49
Non-white	5.26**	4.71 to 5.81
Country of birth		
UK	4.39	4.29 to 4.49
Elsewhere	4.80*	4.43 to 5.18
Marital status		
Married	3.68	3.56 to 3.79
Living as a couple	3.89	3.38 to 4.41
Single never married	6.36***	5.95 to 6.77
Separated or divorced	5.78***	5.54 to 6.03
Widowed	5.75***	5.54 to 5.95
Job status		
In work	3.77	3.58 to 3.96
Not in work	4.51***	4.40 to 4.62
Education		
Degree	3.07	2.87 to 3.27
Other higher	3.60**	3.36 to 3.84
A level	3.93***	3.70 to 4.16
GCSE	3.68***	3.46 to 3.90
Other	4.71***	4.50 to 4.92
None	5.41***	5.23 to 5.59
Social class		
Man and Prof	3.61	3.46 to 3.77
Intermediate	4.22***	4.00 to 4.44
Small emp. and own acc.	4.48***	4.16 to 4.80
Lower supervisory and tech.	4.80***	4.51 to 5.10
Semiroutine and routine	5.09***	4.93 to 5.26
Never had a job	4.65***	4.25 to 5.05
Government office region		
South East	3.93	3.64 to 4.22
North East	4.66**	4.30 to 5.02
North West	4.61**	4.28 to 4.94
Yorkshire and the Humber	4.41*	4.13 to 4.69
East Midlands	4.80***	4.45 to 5.15
West Midlands	4.57**	4.22 to 4.91
East of England	4.16	3.96 to 4.36
London	4.40*	4.09 to 4.71
South West	4.22	3.90 to 4.54
Scotland	4.59*	4.17 to 5.01
Wales	4.72**	4.37 to 5.07
Northern Ireland	4.76**	4.22 to 5.29
Area type		
Urban	4.41	4.29 to 4.53
Rural	4.43	4.24 to 4.61
Car access		
Yes	3.92	3.83 to 4.02
No	6.30***	6.07 to 6.53
Mobile phone		
Yes	4.09	3.99 to 4.19
No	5.50***	5.27 to 5.73
Internet use		
Often	3.41	3.29 to to 3.54
Sometimes	3.77**	3.55 to 3.99
Never	5.14***	5.01 to 5.27

unweighted N; weighted means.

Linear regression models test for significance of mean differences: * p<0.05, ** p<0.01, *** p<0.001.

GHQ, 12 item General Health Questionnaire; LLTI, limiting long-term illness/disability; SEI, Social Exclusion Index; SRH, self-rated health.

The results for the linear regression models predicting SEI by the three health transition measures are shown in table 2. The association between SRH and SEI after adjustment for age and gender could still be seen (model 0) and suggests a dose–response relationship with similar increases in SEI (≈ 1 point) for transitions from good to poor and poor to good SRH and a larger increase (≈ 2) for those with poor SRH at both time points. There was some attenuation in the relationships after controlling for the hypothesised confounders (model 1) and more limited attenuation after including the set of potential mediators (area type, car access, mobile phone ownership and internet use). Model 3 confirmed an independent relationship between SRH and the SEI after accounting for the other health transition measures (LLTI and GHQ).

**Table 2:** Linear regression estimates and 95% CIs for the Social Exclusion Index (Wave 2/3) regressed on health transitions (Wave 1-2)

	Model 0	Model 1	Model 2	Model 3
SRH				
Stable good	0.00 Reference	0.00 Reference	0.00 Reference	0.00 Reference
Good→poor	1.06*** (0.77 to 1.34)	0.83*** (0.56 to 1.10)	0.76*** (0.49 to 1.02)	0.58*** (0.30 to 0.86)
Poor→good	1.10*** (0.79 to 1.41)	0.81*** (0.52 to 1.10)	0.76*** (0.47 to 1.04)	0.61*** (0.32 to 0.90)
Stable poor	1.81*** (1.59 to 2.03)	1.39*** (1.19 to 1.60)	1.31*** (1.11 to 1.51)	0.95*** (0.72 to 1.18)
LLTI				
Stable no LLTI	0.00 Reference	0.00 Reference	0.00 Reference	0.00 Reference
LLTO onset	0.45** (0.17 to 0.74)	0.31* (0.07 to 0.56)	0.30* (0.05 to 0.54)	0.00 (−0.25 to 0.24)
LLTI recovery	0.51*** (0.25 to 0.78)	0.37** (0.13 to 0.62)	0.33** (0.10 to 0.56)	0.05 (−0.17 to 0.28)
Stable LLTI	1.30*** (1.10 to 1.49)	0.96*** (0.78 to 1.14)	0.91*** (0.74 to 1.09)	0.22* (0.02 to 0.42)
GHQ				
Stable low	0.00 Reference	0.00 Reference	0.00 Reference	0.00 Reference
Low→high	0.73*** (0.40 to 1.06)	0.58*** (0.30 to 0.86)	0.60*** (0.32 to 0.88)	0.28* (0.01 to 0.54)
High→low	0.67*** (0.38 to 0.97)	0.54*** (0.27 to 0.82)	0.52*** (0.25 to 0.78)	0.27 (0.00 to 0.54)
Stable high	1.59*** (1.27 to 1.91)	1.41*** (1.13 to 1.70)	1.41*** (1.15 to 1.68)	0.91*** (0.64 to 1.18)

Model 0: Baseline model adjusted for Wave 1 gender, age and age^2^.

Model 1: M0+Wave 1 controls (ethnicity, UK born, marital status, job status, education, social class, region).

Model 2: M1+Wave 1 mediators (urban/rural, car access, mobile phone ownership, internet use).

Model 3: M2+other Wave 1-2 health transition measures.

*p<0.05, **p<0.01,***p<0.001.

GHQ, 12 item General Health Questionnaire; LLTI, limiting long-term illness/disability; SEI, Social Exclusion Index; SRH, self-rated health.

The regression coefficients for LLTI transitions in model 0 showed similar trends in the LLTI to SEI relationship, although the magnitude of the differences in SEI was smaller than that seen for SRH. Again, there was some attenuation after accounting for the confounders but little further change after adjusting for the mediators. In model 3, the coefficients were reduced further and only stable LLTI was associated with an increase in SEI (b=0.22).

GHQ transitions were also related to social exclusion: SEI scores were raised for those with high GHQ at one or 2 time points. Independent of the confounders, mediators and other health measures, only stable high GHQ and a transition from low to high GHQ remained predictive of higher SEI scores.

### Modification of social exclusion and prior health relationship

**Figure 1:**
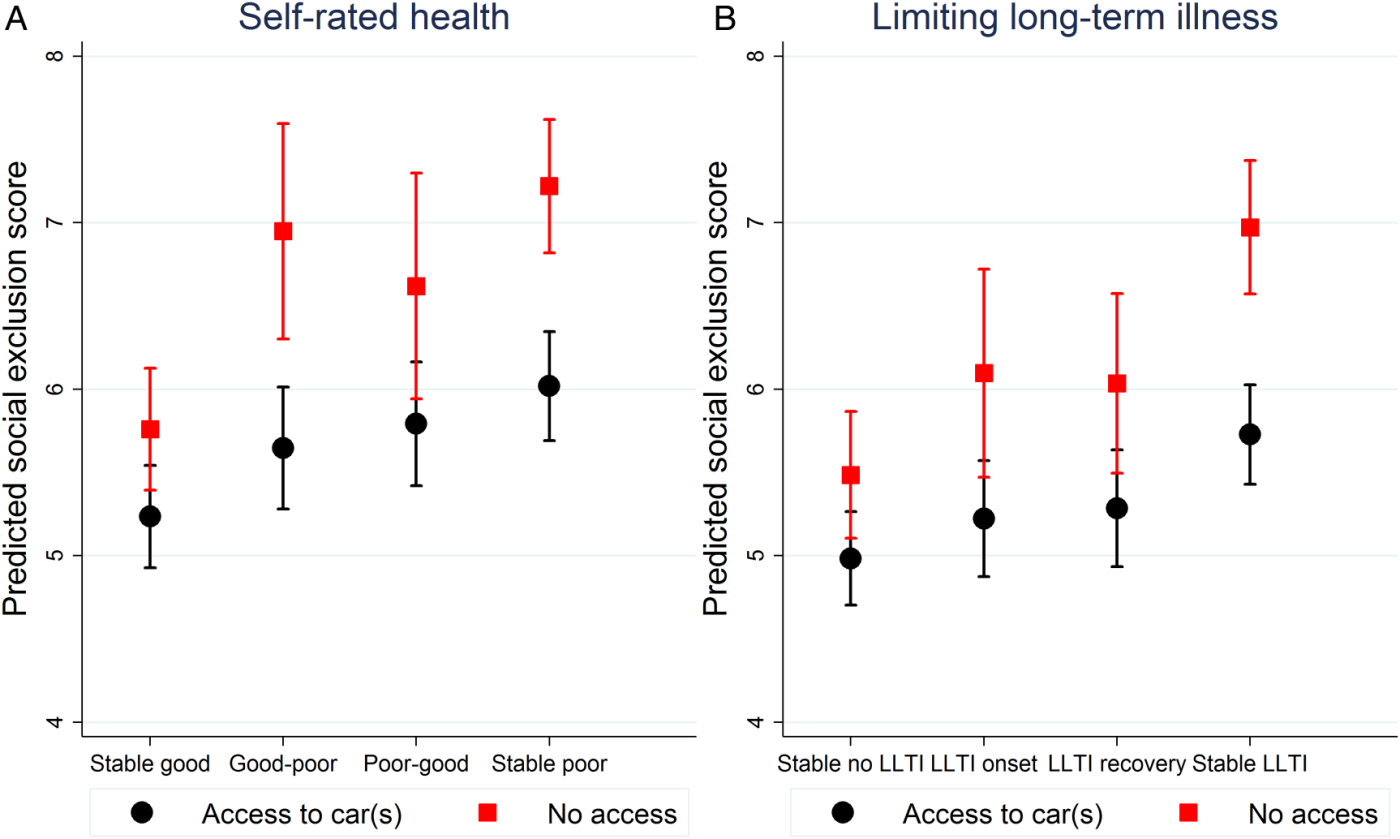
Modification of relationship between health transitions Wave 1-2 and the Social Exclusion Index in Wave 2/3 by access to car(s) in Wave 1. LLTI, limited long-term illness/disability.

We investigated whether there was any moderation of the association between health transitions and SEI by car access, mobile phone ownership and internet use. There was a significant interaction between car access and SRH transitions (p=0.007) and between car access and LLTI transitions (p=0.03): Car access made no difference to SEI scores for those in stable good health but respondents who transitioned to poor health (SRH only) or had stable poor health had lower scores on the SEI when they had access to a car than when not (see figure 1).

**Figure 2:**
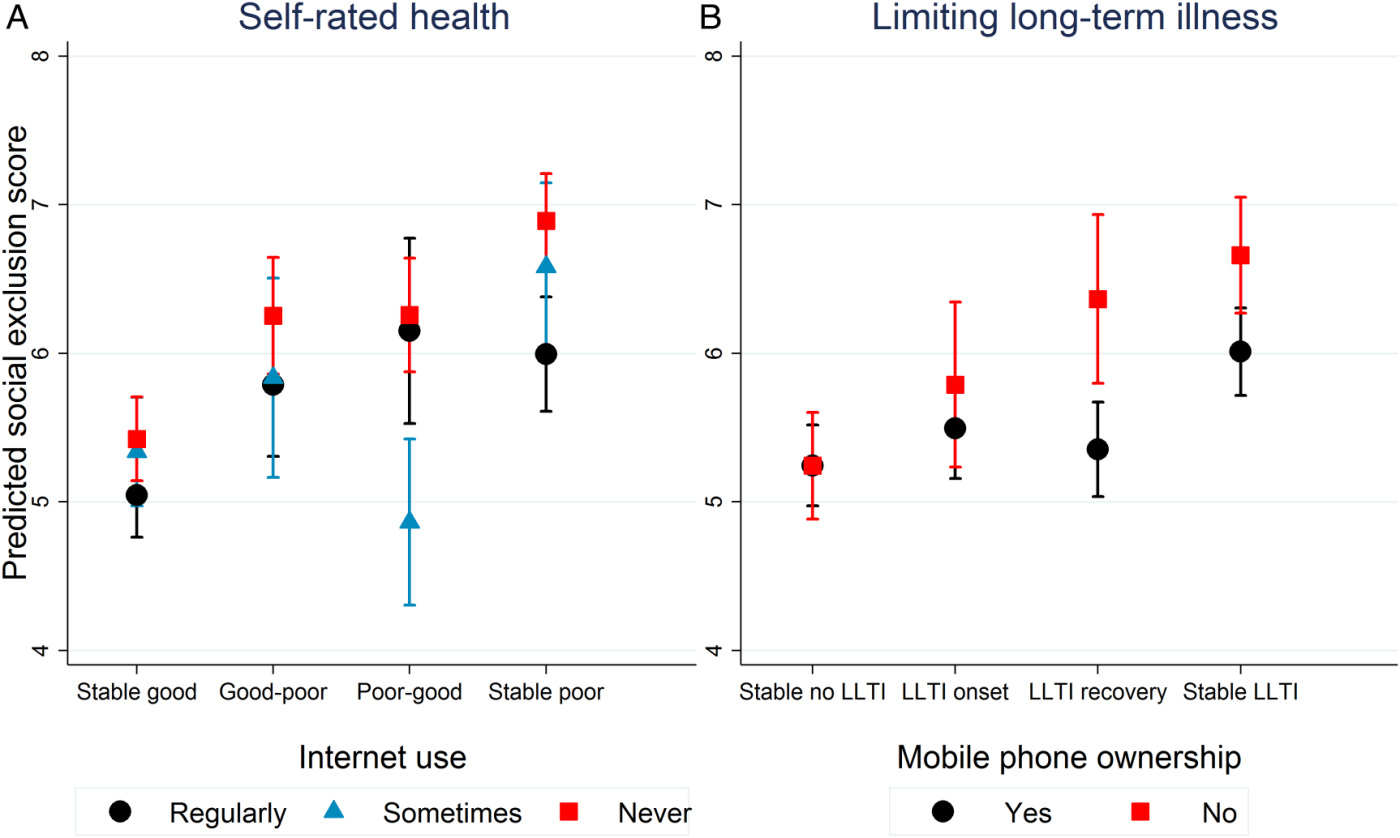
Modification of relationship between health transitions Wave 1-2 and the Social Exclusion Index in Wave 2/3 by technology use in Wave 1. LLTI, limited long-term illness/disability.

Modification of the SRH and SEI relationship by internet use (p=0.003) and of the LLTI and SEI relationship by mobile phone ownership (p=0.0001) was also observed. Again, no difference in SEI scores was observed for those in stable good health. However, respondents with stable poor SRH had higher scores on the SEI if they never used the internet than if they did (figure 2A) and SEI scores were higher for respondents with stable LLTI without a phone than with (figure 2B). Furthermore, occasional internet use was associated with lower social exclusion scores than regular use for those whose SRH improved and having a mobile phone was associated with lower social exclusion scores for those no longer reporting LLTI.

Unlike the other two health transition measures, there was no evidence of moderation of the GHQ to SEI relationship, although those with no access to a car and stable high GHQ had higher SEI scores than those with access to a car and stable low GHQ (p=0.02).

### Social exclusion subdomains and prior health

Results using the social exclusion subdomains (see online supplementary appendix table A3) indicated a much stronger relationship between SRH and civic participation than between SRH and service provision/access or social relations and resources. For LLTI, associations were strongest for civic participation, then service provision/access and weakest for social relations and resources. Like SRH, GHQ was more strongly associated with civic participation than with the service provision/access and social relations and resources subdomains, although the magnitude of the differences was not so great.

### Social exclusion and subsequent health

Table 3 shows the relationship of the covariates and the SEI with poor health at Wave 4. First, there was a clear gradient in poor health by the SEI for all three health measures. Second, there was confirmation of continuity in poor health in Wave 3 to poor health 1-year later, especially for poor self-rated health and limiting long-term illness. Third, differences in rates of poor health across categories of all the covariates were found for at least one of the three health outcomes with the exception of country of birth. For consistency across health measures, we include the same set of covariates in the modelling of the relationship between social exclusion and subsequent health (table 4).

**Table 3:** Weighted per cent (95% CI) in poor health at Wave 4 by explanatory factors (N=4244)

	Poor SRH	LLTI	High GHQ
Wave 2/3			
*SEI*			
0—5	23.77 (21.99 to 25.66)	34.03 (31.98 to 36.14)	13.53 (12.03 to 15.17)
6—10	47.64*** (44.31 to 50.98)	51.51*** (48.33 to 54.67)	21.92*** (19.27 to 24.83)
11—15	72.22*** (44.31 to 50.98)	78.67*** (63.76 to 88.54)	42.68*** (27.24 to 59.69)
Wave 1			
*Gender*			
Male	31.58 (29.24 to 34.01)	37.72 (35.20 to 40.30)	12.45 (10.89 to 14.20)
Female	32.04 (29.76 to 34.41)	42.05* (39.70 to 44.43)	19.99*** (18.04 to 22.09)
Age (years)			
<75	25.56 (23.85 to 27.34)	35.38 (33.51 to 37.31)	14.85 (13.44 to 16.37)
≥75	30.30*** (27.09 to 33.72)	50.37*** (46.74 to 54.00)	20.26** (17.41 to 23.44)
Ethnicity			
White	31.60** (29.89 to 33.37)	40.14 (38.33 to 41.98)	16.50 (15.12 to 17.98)
Non-white	41.29 (34.72 to 48.19)	36.82 (29.65 to 44.63)	18.01 (13.08 to 24.28)
Country of birth			
UK	31.69 (29.94 to 33.50)	40.29 (38.43 to 42.18)	16.49 (15.07 to 18.00)
Elsewhere	34.10 (28.75 to 39.89)	36.21 (30.71 to 42.10)	17.42 (13.97 to 21.50)
Education			
Degree	20.15 (16.79 to 23.99)	33.40 (29.13 to 37.96)	13.57 (10.83 to 16.88)
Other higher	22.36 (18.11 to 27.27)	34.47 (29.61 to 39.67)	13.87 (10.65 to 17.87)
A level	31.89*** (27.55 to 36.58)	35.40 (30.89 to 40.19)	14.57 (11.54 to 18.23)
GCSE	27.11* (22.94 to 31.73)	32.69 (28.57 to 37.10)	16.48 (13.19 to 20.41)
Other	32.74*** (29.08 to 36.63)	42.68** (38.50 to 46.97)	17.28 (14.54 to 20.42)
None	41.05*** (37.75 to 44.43)	47.77*** (44.44 to 51.11)	18.96* (16.51 to 21.68)
Wave 3			
*Poor SRH*			
No	13.01 (11.62 to 14.54)	26.01 (24.07 to 28.05)	10.70 (9.51 to 12.03)
Yes	75.89*** (73.10 to 78.48)	72.98*** (69.92 to 75.83)	30.21*** (27.15 to 33.45)
LLTI			
No	12.02 (10.31 to 13.97)	13.92 (12.14 to 15.91)	10.64 (9.19 to 12.29)
Yes	47.68*** (45.22 to 50.15)	60.98*** (58.67 to 63.25)	21.26*** (19.32 to 23.33)
High GHQ			
No	27.99 (26.25 to 29.79)	36.33 (34.47 to 38.24)	10.88 (9.75 to 12.13)
Yes	60.16*** (55.13 to 64.98)	67.59*** (62.86 to 71.98)	58.29*** (53.02 to 63.38)
Marital status			
Married	28.18 (26.12 to 30.33)	35.90 (33.64 to 38.23)	15.27 (13.56 to 17.15)
Living as a couple	22.53 (14.31 to 33.64)	41.70 (34.37 to 49.42)	11.15 (5.74 to 20.55)
Single never married	37.58* (30.21 to 45.57)	48.60 (43.32 to 53.92)	17.67 (13.42 to 22.90)
Separated or divorced	37.42** (32.49 to 42.63)	47.79*** (43.93 to 51.68)	18.68 (14.88 to 23.19)
Widowed	38.74*** (35.11 to 42.51)	33.08*** (23.75 to 43.96)	19.28* (16.28 to 22.67)
Job status			
In work	14.27 (10.77 to 18.66)	20.16 (15.92 to 25.18)	7.83 (5.49 to 11.05)
Not in work	33.51*** (31.72 to 35.36)	41.97*** (40.07 to 43.90)	17.37*** (15.93 to 18.92)
Social class			
Man and Prof	24.81 (22.14 to 27.69)	35.46 (32.39 to 38.64)	14.18 (12.16 to 16.48)
Intermediate	29.39 (25.32 to 33.82)	39.42 (34.98 to 44.04)	19.64* (16.13 to 23.70)
Small emp. and own acc.	27.84*** (22.38 to 34.06)	37.76 (32.10 to 43.77)	18.68 (14.33 to 23.99)
Lower supervisory and tech.	40.50*** (34.17 to 47.16)	50.10*** (43.66 to 56.54)	16.01 (11.70 to 21.53)
Semiroutine and routine	38.45 (35.51 to 41.48)	43.34** (40.28 to 46.44)	17.35* (15.17 to 19.76)
Never had a job	36.19** (28.57 to 44.58)	39.97 (32.02 to 48.48)	14.58 (10.01 to 20.74)
Region			
South East	26.19 (21.88 to 31.01)	37.72 (32.99 to 42.69)	15.98 (12.25 to 20.57)
North East	35.58* (27.83 to 44.17)	42.04 (32.74 to 51.94)	17.93 (11.45 to 26.95)
North West	32.78 (27.43 to 38.62)	40.99 (35.74 to 46.45)	14.75 (11.30 to 19.04)
Yorkshire and the Humber	34.17* (28.38 to 40.47)	39.79 (33.81 to 46.10)	15.93 (11.83 to 21.13)
East Midlands	33.77 (27.71 to 40.42)	41.50 (35.01 to 48.30)	17.60 (13.50 to 22.63)
West Midlands	31.28 (26.80 to 36.14)	36.87 (31.51 to 42.57)	21.96 (17.13 to 27.69)
East of England	31.53 (26.78 to 36.70)	42.43 (37.36 to 47.67)	15.19 (11.85 to 19.28)
London	29.80 (24.32 to 35.93)	33.31 (28.16 to 38.89)	21.50 (17.30 to 26.38)
South West	28.12 (23.24 to 33.57)	40.39 (34.54 to 46.52)	12.37 (9.28 to 16.32)
Wales	42.15*** (36.00 to 48.56)	47.43* (39.52 to 55.46)	17.30 (11.50 to 25.18)
Scotland	35.40* (29.58 to 41.68)	40.87 (35.73 to 46.22)	17.51 (13.20 to 22.84)
Northern Ireland	33.43 (23.87 to 44.59)	41.40 (30.48 to 53.23)	12.85 (7.13 to 22.05)
Area type			
Urban	33.86 (31.86 to 35.92)	42.00 (39.91 to 44.13)	17.98 (16.32 to 19.77)
Rural	27.19 (24.15 to 30.46)	35.66** (32.43 to 39.02)	13.26** (11.15 to 15.71)
Car access			
Yes	28.78 (26.93 to 30.70)	36.67 (34.73 to 38.64)	15.10 (13.66 to 16.67)
No	43.00 (39.14 to 46.94)	52.52*** (48.55 to 56.46)	21.80*** (18.81 to 25.13)
Mobile phone			
Yes	29.79 (28.03 to 31.61)	37.72 (35.85 to 39.62)	15.88 (14.46 to 17.42)
No	40.83 (36.50 to 45.30)	50.45*** (45.95 to 54.94)	19.43* (16.17 to 23.17)
Internet use			
Often	22.78 (20.70 to 25.00)	32.16 (29.86 to 34.55)	13.73 (12.07 to 15.57)
Sometimes	29.52* (24.88 to 34.63)	37.93* (32.74 to 43.41)	13.65 (10.39 to 17.72)
Never	40.20*** (37.53 to 42.94)	47.41*** (44.64 to 50.19)	19.60*** (17.51 to 21.87)

Logistic regression models test for difference in proportions: * p<0.05, ** p<0.01,*** p<0.001.

GHQ, 12 item General Health Questionnaire; LLTI, limited long-term illness/disability; poor SRH, fair/poor self-rated health; SEI, Social Exclusion Index.

**Table 4:** Logistic regression estimates (ORs and 95% CIs) for poor health outcomes in Wave 4 regressed on the Social Exclusion Index at Waves 2/3

	Model 0	Model 1	Model 2
Poor SRH	1.16*** (1.11 to 1.21)	1.14*** (1.09 to 1.20)	1.15*** (1.09 to 1.21)
LLTI	1.08*** (1.04 to 1.12)	1.08** (1.03 to 1.13)	1.07** (1.02 to 1.12)
High GHQ	1.04 (0.99 to 1.09)	1.07** (1.02 to 1.13)	1.07* (1.02 to 1.13)

Model 0: Baseline model adjusted for Wave 3 SRH, LLTI, GHQ, gender, age and age^2^.

Model 1: M0+Wave 1 controls (ethnicity, UK born, education)+Wave 3 controls (marital status, job status, social class, region).

Model 2: M1+Wave 3 mediators (urban/rural, car access, mobile phone ownership, internet use).

* p<0.05, ** p<0.01, *** p<0.001.

GHQ, 12 item General Health Questionnaire; LLTI, limited long-term illness/disability; SRH, self-rated health.

The baseline model 0 adjusted for health in Wave 3, age and gender. There was a 16% increase in the odds of poor SRH for each unit increase in the SEI conditional on health at Wave 3. After adjustment for the control variables in model 1, the odds were essentially unchanged. The potential mediators did not attenuate this association between social exclusion and subsequent health.

A similar set of results can be seen for limiting long-term illness (table 4, second row). The odds of LLTI in Wave 4 were increased by 8% for each unit increase in the SEI. This remained unchanged with the addition of the control variables and the hypothesised mediators. For the GHQ baseline model, the odds were 1.04 per unit increase in the SEI. However, these odds were revealed to be slightly higher and statistically significant after the adjustments in models 1 and 2.

There was no evidence of any moderation of the positive association between poor health and subsequent SEI by area type, car access, mobile phone ownership or internet use. The subdomain analyses (see online supplementary appendix table A4) indicated that conditional on health in Wave 3, social exclusion in the domains of civic participation and social relations and resources increased the probability of poor SRH. The domains of civic participation and service provision and access, but not social relations and resources, were related to subsequent LLTI. GHQ was associated with prior civic participation only.

## Discussion

This study adopted a new analytical framework for understanding health and social exclusion in older age by explicitly separating measures of health from those of social exclusion. Previous work has confounded the two concepts making it difficult to understand the dynamics between health and social exclusion. Using this approach, we have both confirmed and extended existing knowledge on social exclusion in older age. Consistent with expectations, we found that poor health predicted social exclusion 1–2 years later. Given the health status at baseline, we also found that social exclusion predicted later declines in health. Finally, we identified use of a car, mobile phone and the internet as factors that might support older adults in poor health and help break the downward spiral in well-being.

We failed to find much evidence that improvements in health reduced social exclusion in the short term. Once data are available, it should be possible to elucidate the longer term dynamics hinted at by the observed differences in SEI scores across 1-year health transition categories. Somewhat surprisingly, there was no evidence that urban/rural location, car access, mobile phone ownership or internet use explained the prior health with later SEI relationship, even though both health and SEI were related to each of these potential mediators. There are a number of other mechanisms that might explain the relationship between health and social exclusion that could also be amenable to policy intervention. Examples include the material and financial consequences of poor health,^5^ ^28^ ^37^ discrimination^38^ and environmental factors beyond those considered here.^5^ ^28^ ^38^

The evidence for effect modification also suggests points for intervention. The role of car access suggests that alternatives to the car, such as improved public transport and taxi schemes for the elderly, might also be able to prevent social exclusion. The importance of internet use and technology highlights the need for further research to understand which capabilities constrain older adults' use of these forms of communication. There was no modification of the relationship between GHQ and social exclusion. On the one hand, this is unsurprising since symptoms of depression and anxiety include diminished interest and loss of pleasure in social activities.^39^ On the other, this distinction between physical and psychological health may provide a clue that it is physical and cognitive capabilities, rather than psychosocial capabilities, driving use of the internet and technology. The finding that occasional internet use was more beneficial than regular use for respondents whose SRH improved might also suggest that those in poor health were seeking information online.

Civic participation was the subdomain most strongly and consistently associated with health, both as an outcome of health transitions and as a predictor of subsequent health change. That there is a bidirectional association is consistent with findings from Europe,^13^ but our findings go further in showing the dominance of civic participation over service provision and access and social relations and resources. Another study found that over 20% of Canadian seniors wanted to be more involved in social activities,^40^ highlighting the extent of need in this area.

Our study has some distinct strengths: we used data from a large contemporary panel study, which meant that we were able to take advantage of the longitudinal design to investigate the relationship between health and social exclusion unfolding over time; we tested a new analytical framework for our analysis; and we considered multiple domains of health and social exclusion. On the other hand, some limitations must be acknowledged. First, as in all longitudinal studies, there were missing data which may have affected our results. The vast majority of missing data was for the GHQ-12 scores as these were completed mainly as part of a self-completion module. We repeated the analyses for those with complete data over Waves 1–4 (not shown); the substantive findings were unchanged but lacked precision due to the smaller sample size, so we present results using complete cases for each research question. We used longitudinal weights to account for dropout. Any bias introduced by non-response is likely to have underestimated effects. Second, the SEI index is specific to Understanding Society and of necessity its construction was limited by the data available, although many government departments and third sector agencies rely on these data for evidence-based policy development. Nevertheless, the SEI may not have fully captured all dimensions of the subdomains. Finally, Walsh *et als*' framework that guided our analysis was conceptualised for a specific rural context and may not generalise to the UK population.

## Conclusion

There is synergy between health and social exclusion among older people living independently in the UK. Our findings suggest that it might be more effective to target the prior health to exclusion relationship than the exclusion to later health relationship. Designing age-friendly hardware and software might support social inclusion in later life.

**What is already known on this subject**

- Poor health and social exclusion cluster in older adults but the causal mechanism is less clear.

**What this study adds**

- Among non-community living older people in the UK, poor health is associated with greater social exclusion and, in turn, social exclusion is linked to health decline. Use of a car, mobile phone and the internet are factors that protected older adults in poor health from social exclusion. Designing age-friendly hardware and software might have public health benefits.
