# Supplementary material for: Health and social exclusion in older age: evidence from Understanding Society, the UK household longitudinal study
Source: J Epidemiol Community Health. 2017 Feb 22;71(7):681–90. doi: 10.1136/jech-2016-208037 (PMC5485754; doi:10.1136/jech-2016-208037)
Supplement: supplementary appendix [file jech-2016-208037supp001.pdf]

Appendix

Table A1 Descriptive statistics for excluded cases and complete case sample for the regression of Social Exclusion Index (wave 2/3) on health (wave 1)

		Excluded cases		Complete cases		p
		N	Mean or %	N	Mean or %	
**Wave 1**						
Age (mean)		1163	74.11	4312	71.73	<0.0001
Gender (%)	Male	536	44.08	1981	45.86	0.33
	Female	627	55.92	2331	54.14	
Ethnicity (%)	White	1018	93.53	4167	97.95	<0.0001
	Non-white	139	6.47	145	2.05	
Country of birth (%)	UK	980	88.53	4042	94.82	0.0001
	Elsewhere	180	11.47	270	5.18	
Education (%)	Degree	124	9.88	566	11.99	<0.0001
	Other higher	107	8.16	522	10.89	
	A level	102	8.51	530	11.73	
	GCSE	104	8.07	602	12.58	
	Other	199	17.76	757	18.20	
	None	523	47.64	1335	34.60	
Poor SRH (%)	No	762	62.11	3139	71.40	<0.0001
	Yes	469	37.89	1209	28.60	
LLTI (%)	No	631	52.35	2629	59.47	0.0008
	Yes	547	47.65	1719	40.53	
High GHQ (%)	No	395	82.79	3638	83.36	0.79
	Yes	83	17.21	710	16.64	
Marital status (%)	Married	604	53.06	2725	63.30	<0.0001
	Living as a couple	30	2.60	127	2.83	
	Single never married	71	5.81	187	4.36	
	Separated or divorced	118	8.20	417	8.15	
	Widowed	338	30.34	856	21.36	
Job status (%)	In work	124	9.45	593	13.23	0.004
	Not in work	1037	90.55	3719	86.77	
Social class (%)	Man & Prof	274	25.21	1391	30.55	0.004
	Intermediate	124	10.91	591	13.46	
	Small emp. & own acc.	110	9.38	409	9.77	
	Lower supervisory/ tech	80	8.03	342	8.19	
	Semi-routine & routine	390	38.63	1345	32.28	
	Never had a job	94	7.84	234	5.76	
Region (%)	South East	161	14.29	632	15.42	<0.0001
	North East	47	4.47	184	4.23	
	North West	114	9.50	502	11.71	
	Yorkshire & Humber	119	10.39	342	7.95	
	East Midlands	74	5.61	372	7.69	

		Excluded cases		Complete cases		p
		**N**	**Mean or %**	N	**Mean or %**	
	West Midlands	105	9.02	348	8.49	
	East of England	98	8.35	451	10.40	
	London	135	12.72	229	6.13	
	South West	93	7.72	482	10.58	
	Wales	79	6.58	276	5.96	
	Scotland	62	6.93	336	9.14	
	Northern Ireland	92	4.44	169	2.29	
Area type (%)	Urban	853	75.25	2918	69.43	0.006
	Rural	310	24.75	1394	30.57	
Car access (%)	Yes	819	69.72	3512	79.44	<0.0001
	No	336	30.28	800	20.56	
Mobile phone (%)	Yes	797	64.46	3448	76.91	<0.0001
	No	358	35.54	864	23.09	
Internet use (%)	Often	267	21.37	1557	32.93	<0.0001
	Sometimes	128	9.55	520	11.46	
	Never	760	69.08	2235	55.62	
**Wave 2/3**						
SEI (mean)		1163	5.27	4312	4.41	<0.0001

Note: unweighted N; weighted means

SRH – self rated health; LLTI – limited long-term illness/disability; GHQ – 12 item General Health Questionnaire

Pearson Chi-square test for significant difference in proportions and linear regression models test for significant difference in means for complete vs. excluded case

Table A2 Descriptive statistics for excluded cases and complete case sample for the regression of on health (wave 4) on the Social Exclusion Index (wave 2/3)

		**Excluded cases**		**Complete cases**		**p**
		**N**	**Mean or %**	N	**Mean or %**	
**Wave 1**						
Age (mean)		1231	74.68	4244	71.49	<0.0001
Gender (%)	Male	562	44.44	1955	45.79	0.45
	Female	669	55.56	2289	54.21	
Ethnicity (%)	White	1117	94.68	4068	97.69	<0.0001
	Non-white	108	5.32	176	2.31	
Country of birth (%)	UK	1077	90.37	3945	94.40	0.0002
	Elsewhere	151	9.63	299	5.60	
Education (%)	Degree	88	5.98	602	13.30	<0.0001
	Other higher	114	8.17	515	10.96	
	A level	97	7.99	535	11.98	
	GCSE	108	7.79	598	12.79	
	Other	215	17.77	741	18.20	
	None	605	52.31	1253	32.76	
**Wave 2/3**						
SEI (mean)		1231	5.52	4244	4.31	<0.0001
**Wave 3**						
Poor SRH (%)	No	718	57.53	3024	70.08	<0.0001
	Yes	510	42.47	1220	29.92	
LLTI (%)	No	473	37.12	1898	44.45	0.0007
	Yes	757	62.88	2346	55.55	
High GHQ-12 (%)	No	315	81.95	3743	88.07	0.01
	Yes	66	18.05	501	11.93	
Marital status (%)	Married	706	57.10	2542	59.87	0.06
	Living as a couple	27	1.98	113	2.59	
	Single never married	59	4.42	203	4.87	
	Separated or divorced	105	7.28	429	8.53	
	Widowed	334	29.22	957	24.13	
Job status (%)	In work	65	4.68	387	8.76	0.0001
	Not in work	1166	95.32	3857	91.24	
Social class (%)	Man & Prof	257	20.94	1403	31.95	<0.0001
	Intermediate	118	9.97	592	13.70	
	Small emp. & own acc.	114	9.59	421	10.01	
	Lower supervisory/ tech	102	9.36	314	7.65	
	Semi-routine & routine	449	41.22	1295	31.47	
	Never had a job	102	8.92	219	5.22	
Region (%)	South East	129	11.83	648	16.36	<0.0001
	North East	36	3.53	189	4.53	
	North West	109	8.91	505	11.86	
	Yorkshire & Humber	148	12.00	314	7.39	

		Excluded cases		Complete cases		p
		**N**	**Mean or %**	N	**Mean or %**	
	East Midlands	130	9.21	317	6.63	
	West Midlands	125	10.43	327	8.05	
	East of England	112	9.09	439	10.24	
	London	106	10.29	252	6.52	
	South West	65	5.66	512	11.37	
	Wales	92	7.25	262	5.80	
	Scotland	65	5.66	512	11.37	
	Northern Ireland	92	7.25	262	5.80	
Area type (%)	Urban	879	74.30	2888	69.46	0.04
	Rural	352	25.70	1356	30.54	
Car access (%)	Yes	834	65.11	3408	78.56	<0.0001
	No	396	34.89	836	21.44	
Mobile phone (%)	Yes	374	35.10	659	18.43	<0.0001
	No	856	64.90	3585	81.57	
Internet use (%)	Often	243	16.80	1911	41.85	<0.0001
	Sometimes	98	7.76	459	10.16	
	Never	890	75.44	1874	47.99	
**Wave 4**						
Poor SRH (%)	No	671	53.48	2946	68.17	<0.0001
	Yes	559	46.52	1298	31.83	
LLTI (%)	No	589	46.30	2594	59.94	<0.0001
	Yes	639	53.70	1650	40.06	
High GHQ (%)	No	1792	81.78	3612	83.46	0.46
	Yes	406	18.22	673	16.54	

Note: unweighted N; weighted means
SRH – self rated health; LLTI – limited long-term illness/disability; GHQ – 12 item General Health Questionnaire
Pearson Chi-square test for significant difference in proportions and linear regression models test for significant difference in means for complete vs. excluded cases

Table A3. Linear regression estimates and 95% confidence intervals for the Social Exclusion subscales (wave 2/3) regressed on health (wave 1)

		Model 0	Model 1	Model 2	Model 3
		**Civic participation**			
SRH	Stable good	0.00	0.00	0.00	0.00
		[ref]	[ref]	[ref]	[ref]
	Good→poor	0.61***	0.47***	0.43***	0.39***
		[0.44, 0.78]	[0.31, 0.63]	[0.27, 0.58]	[0.23, 0.56]
	Poor→good	0.60***	0.41***	0.37***	0.33***
		[0.41, 0.79]	[0.23, 0.59]	[0.19, 0.55]	[0.15, 0.51]
	Stable poor	1.00***	0.74***	0.69***	0.60***
		[0.88, 1.10]	[0.62, 0.87]	[0.57, 0.81]	[0.46, 0.74]
LLTI	Stable no LLTI	0.00	0.00	0.00	0.00
		[ref]	[ref]	[ref]	[ref]
	LLTI onset	0.27**	0.18*	0.16*	-0.01
		[0.10, 0.43]	[0.03, 0.33]	[0.01, 0.31]	[-0.15, 0.14]
	LLTI recovery	0.26**	0.16	0.14	-0.01
		[0.08, 0.43]	[0.00, 0.32]	[-0.02, 0.29]	[-0.16, 0.14]
	Stable LLTI	0.60***	0.44***	0.41***	0.02
		[0.49, 0.72]	[0.33, 0.55]	[0.30, 0.52]	[-0.10, 0.14]
GHQ	Stable low	0.00	0.00	0.00	0.00
		[ref]	[ref]	[ref]	[ref]
	Low→high	0.31**	0.18*	0.19*	0.01
		[0.12, 0.50]	[0.01, 0.34]	[0.02, 0.35]	[-0.16, 0.17]
	High→low	0.36***	0.25**	0.24**	0.11
		[0.19, 0.53]	[0.09, 0.42]	[0.08, 0.40]	[-0.05, 0.27]
	Stable high	0.77***	0.64***	0.62***	0.35***
		[0.58, 0.97]	[0.46, 0.82]	[0.46, 0.79]	[0.18, 0.53]
		**Service provision/access**			
SRH	Stable good	0.00	0.00	0.00	0.00
		[ref]	[ref]	[ref]	[ref]
	Good→poor	0.31***	0.29***	0.27***	0.16*
		[0.18, 0.43]	[0.16, 0.42]	[0.14, 0.39]	[0.03, 0.29]
	Poor→good	0.22**	0.19*	0.20**	0.12
		[0.07, 0.36]	[0.05, 0.34]	[0.06, 0.35]	[-0.03, 0.26]
	Stable poor	0.42***	0.39***	0.38***	0.20**
		[0.33, 0.52]	[0.29, 0.49]	[0.29, 0.48]	[0.09, 0.32]
LLTI	Stable no LLTI	0.00	0.00	0.00	0.00
		[ref]	[ref]	[ref]	[ref]
	LLTI onset	0.19**	0.16**	0.17**	0.09
		[0.07, 0.30]	[0.05, 0.28]	[0.05, 0.28]	[-0.03, 0.20]
	LLTI recovery	0.20*	0.19**	0.18**	0.12
		[0.09, 0.31]	[0.08, 0.30]	[0.08, 0.29]	[0.01, 0.22]
	Stable LLTI	0.39***	0.35***	0.36***	0.19
		[0.31, 0.48]	[0.27, 0.44]	[0.27, 0.44]	[0.08, 0.29]
GHQ	Stable low	0.00	0.00	0.00	0.00

		Model 0	Model 1	Model 2	Model 3
		[ref]	[ref]	[ref]	[ref]
	Low→high	0.31***	0.29***	0.30***	0.19
		[0.17, 0.45]	[0.15, 0.43]	[0.16, 0.43]	[0.06, 0.32]
	High→low	0.12	0.12	0.11	0.03
		[-0.01, 0.26]	[-0.02, 0.25]	[-0.02, 0.24]	[-0.10, 0.16]
	Stable high	0.41***	0.39***	0.41***	0.24
		[0.25, 0.56]	[0.24, 0.53]	[0.26, 0.55]	[0.10, 0.39]
		**Social relations and resources**			
SRH	Stable good	0.00	0.00	0.00	0.00
		[ref]	[ref]	[ref]	[ref]
	Good→poor	0.14*	0.07	0.06	0.03
		[0.01, 0.27]	[-0.05, 0.19]	[-0.06, 0.18]	[-0.10, 0.15]
	Poor→good	0.28***	0.21**	0.19**	0.16*
		[0.13, 0.43]	[0.08, 0.33]	[0.07, 0.31]	[0.03, 0.29]
	Stable poor	0.38***	0.26***	0.23***	0.15**
		[0.27, 0.48]	[0.17, 0.35]	[0.15, 0.32]	[0.05, 0.25]
LLTI	Stable no LLTI	0.00	0.00	0.00	0.00
		[ref]	[ref]	[ref]	[ref]
	LLTI onset	0.00	-0.03	-0.03	-0.08
		[-0.12, 0.13]	[-0.13, 0.07]	[-0.13, 0.07]	[-0.19, 0.02]
	LLTI recovery	0.06	0.02	0.01	-0.05
		[-0.07, 0.18]	[-0.08, 0.13]	[-0.09, 0.11]	[-0.16, 0.05]
	Stable LLTI	0.30***	0.17***	0.15***	0.01
		[0.21, 0.40]	[0.09, 0.25]	[0.07, 0.23]	[-0.09, 0.10]
GHQ	Stable low	0.00	0.00	0.00	0.00
		[ref]	[ref]	[ref]	[ref]
	Low→high	0.12	0.12	0.11	0.08
		[-0.03, 0.26]	[0.00, 0.23]	[0.00, 0.23]	[-0.04, 0.20]
	High→low	0.19*	0.17**	0.17**	0.13*
		[0.04, 0.34]	[0.05, 0.30]	[0.04, 0.29]	[0.00, 0.26]
	Stable high	0.41***	0.39***	0.38***	0.32***
		[0.27, 0.56]	[0.25, 0.52]	[0.25, 0.51]	[0.18, 0.45]

SRH – self rated health; LLTI – limiting long-term illness/disability; GHQ – 12 item General Health Questionnaire

Model 0: Baseline model adjusted for gender, age & age^2

Model 1: M0 + controls (ethnicity, UK born, marital status, job status, education, social class, region)

Model 2: M1 + mediators (urban/rural, car access, mobile phone ownership, internet use)

Model 3: M2 + other health measures

* p<0.05, ** p<0.01, *** p<0.001

Table A4. Logistic regression estimates (odds ratios and 95% confidence intervals) for the health outcomes in wave 4 regressed on the Social Exclusion subscales at waves 2/3.

	Model 0	Model 1	Model 2
	**Poor SRH**		
Civic participation	1.29***	1.24***	1.24***
	[1.21,1.39]	[1.15,1.34]	[1.14,1.34]
Service provision/access	1.11*	1.09	1.10
	[1.01,1.22]	[0.98,1.20]	[1.00,1.21]
Social relations and resources	1.14**	1.14*	1.15*
	[1.04,1.24]	[1.01,1.29]	[1.02,1.30]
	**LLTI**		
Civic participation	1.11**	1.09*	1.08*
	[1.04,1.18]	[1.02,1.17]	[1.00,1.16]
Service provision/access	1.12*	1.08	1.10*
	[1.00,1.19]	[0.99,1.18]	[1.01,1.20]
Social relations and resources	1.07	1.08	1.06
	[0.99,1.15]	[0.97,1.19]	[0.96,1.17]
	**High GHQ**		
Civic participation	1.07	1.10*	1.09*
	[0.99,1.16]	[1.01,1.20]	[1.01,1.19]
Service provision/access	1.09	1.10	1.11
	[0.98,1.21]	[0.99,1.22]	[1.00,1.24]
Social relations and resources	1.01	1.05	1.04
	[0.92,1.11]	[0.93,1.19]	[0.92,1.18]

SRH – self rated health; LLTI – limited long-term illness/disability; GHQ – 12 item General Health Questionnaire

Model 0: Baseline model adjusted for SRH (wave 1), LLTI (wave 1), GHQ (wave 1) gender, age & age^2

Model 1: M0 + controls (ethnicity, UK born, marital status, job status, education, social class, region)

Model 2: M1 + mediators (urban/rural, car access, mobile phone ownership, internet use)

* $p<0.05$, ** $p<0.01$, *** $p<0.001$
